# Investigation of the *COMT* Val158Met variant association with age of onset of psychosis, adjusting for cannabis use

**DOI:** 10.1002/brb3.850

**Published:** 2017-10-06

**Authors:** Rohit J. Lodhi, Yabing Wang, David Rossolatos, Georgina MacIntyre, Alexandra Bowker, Candice Crocker, Hongyan Ren, Aleksandra Dimitrijevic, Darren A. Bugbee, Alexandra Loverock, Brett Majeau, Sudhakar Sivapalan, Virginia M. Newton, Philip Tibbo, Scot E. Purdon, Katherine J. Aitchison

**Affiliations:** ^1^ Department of Psychiatry University of Alberta Edmonton AB Canada; ^2^ Department of Medicine University of Alberta Edmonton AB Canada; ^3^ Neuropsychology Alberta Hospital Edmonton AB Canada; ^4^ Department of Psychiatry Dalhousie University Halifax NS Canada; ^5^ Nova Scotia Early Psychosis Program Halifax NS Canada; ^6^ Edmonton Early Intervention in Psychosis Clinic Edmonton AB Canada; ^7^ Department of Medical Genetics University of Alberta Edmonton AB Canada

**Keywords:** cannabis, catechol‐*O*‐methyltransferase, genes, psychotic disorders, sex

## Abstract

**Objective:**

*COMT* rs4680 (Val158Met) genotype moderates the effect of cannabis on the age of onset of psychosis (AoP). We investigated the association between rs4680 and AoP, after adjusting for relevant covariates, in a Canadian Caucasian sample.

**Methods:**

One hundred and sixty‐nine subjects with psychosis were recruited. AoP, defined as age of DSM‐IV diagnosis was established using the Structured Clinical Interview for DSM‐IV. Cannabis use data were collected using a self‐report computerized questionnaire. DNA was extracted from saliva and genotyping of the *COMT* Val158Met polymorphism was done by SNaPshot and TaqMan assays. Logistic regression and Kaplan–Meier analysis results are reported.

**Results:**

In those who had used cannabis before 20 years of age, rs4680 had a trend level effect on AoP (median AoP: Val/Val < Val/Met < Met/Met 19.37, 20.95, 21.24 years, respectively; log‐rank test *p *=* *.051).

**Conclusion:**

Our data are indicative of the need to further investigate the association between the *COMT* rs4680 variant and AoP in the context of adolescent cannabis use.

## 1 INTRODUCTION

Cannabis use is associated with an increased risk of psychosis (Gage, Hickman, & Zammit, 2016). An earlier meta‐analysis reported a 40% increase in risk (95% confidence interval [CI] 20–65%) of any psychotic outcome in cannabis users compared with never users (Moore et al., 2007). In a birth cohort study, cannabis use by age 15 years was associated with increased risk of schizophreniform disorder at age 26 years (Arseneault et al., 2002). In this study, the effect size (OR = 1.65, CI 0.65–4.18) in those first using cannabis by age 18 years was less than that for those first using cannabis by age 15 years (OR = 4.50, CI 1.11–18.21). Although the odds ratios are different, the wide and largely overlapping confidence intervals for these two odds ratios indicate that the risk of psychosis is similar in the two age groups. It has been recommended that further studies be done to identify high‐risk groups particularly susceptible to the effects of cannabis on psychosis (Gage et al., 2016), such as those who are genetically susceptible.

Caspi et al. (2005) reported that a functional variant (rs4680, a G>A substitution that results in a valine to methionine substitution at amino acid codon 158) in the *COMT* gene encoding the enzyme catechol‐*O*‐methyltransferase (COMT) was associated with increased risk of schizophreniform disorder at age 26 years for those with a history of adolescent cannabis use (Caspi et al., 2005). The risk genotype was Val/Val (OR 10.9, CI 2.2–54.1), with the Val/Met being associated with a lesser degree of risk (OR 2.5, CI 0.78–8.2). Consistent with this, others reported a similar gene‐environment (*COMT* rs4680 – cannabis) effect on the age of onset of psychosis (AoP) among individuals within a schizophrenia spectrum disorder (Estrada et al., 2011). In individuals with schizophrenia spectrum disorders, Val/Val genotype had the earliest AoP, followed by Val/Met, followed by Met/Met. Subjects with a first episode of a nonaffective psychosis also exhibit a significant cannabis – rs4680 interaction on AoP as well as on duration of untreated psychosis (Pelayo‐Teran et al., 2010). In a recent publication, however, rs4680 was not associated with AoP, neither independently nor in interaction with another genetic variant that has been previously studied in relation to psychosis and cannabis consumption, namely the *BDNF* Val66Met (Mané et al., 2017). The exact mechanism behind the association between rs4680 and AoP is not clear but cannabis is an important factor affecting AoP (Barnes, Mutsatsa, Hutton, Watt, & Joyce, 2006; Compton et al., 2009; Gonzalez‐Pinto et al., 2008). Other significant factors affecting AoP are gender (Mané et al., 2017) and family history of psychosis/schizophrenia (Byrne, Agerbo, & Mortensen, 2002; Kendler & MacLean, 1990). There have also been reports of associations between rs4680 and other relevant phenotypes, for example, healthy individuals of Val/Val genotype were at increased risk of experiencing hallucinations on cannabis consumption if they had high levels of psychosis vulnerability (Henquet et al., 2009), rs4680 moderating the effect of Δ‐9‐tetrahydrocannbinol on psychosis and cognition (Henquet et al., 2006) and those at risk of transitioning to psychosis showing an increased risk of positive symptoms in the case of Val/Val individuals with a history of cannabis use at least weekly, with a lesser degree of effect for those of Val/Met genotype (Nieman et al., 2016).

To understand the *COMT*‐cannabis‐psychosis/AoP relationship, it is important to consider the following. Dopamine plays a role in psychosis (Howes, McCutcheon, Owen, & Murray, 2017) and in cannabis use (Blum et al., 2017). A genome‐wide association study reported the D_2_ receptor gene (*DRD2*) as one of the 108 loci associated with schizophrenia (Schizophrenia Working Group of the Psychiatric Genomics C, 2014). Dopaminergic agonists and stimulants worsen psychosis (Angrist & Gershon, 1970), while dopamine receptor (D_2_/D_3_) antagonism is key to reducing symptoms of psychosis (Howes et al., 2009; Seeman, Lee, Chau‐Wong, & Wong, 1976). Substance misuse, including cannabis, is linked to reward mechanisms, in which dopamine plays a central role (Blum et al., 2015, 2017). There is significant interaction between dopamine and the endogenous cannabinoid system (ECS) in substance misuse. It has been suggested that the ECS has a significant role to play in the core reward system (Gardner, 2005). The ECS promotes midbrain dopamine cell activation and dopamine release in the nucleus accumbens, thereby facilitating reward behaviors (Wenzel & Cheer, 2017). Cannabis is known to increase dopamine levels in the cortex (Stokes et al., 2010), striatum (Bossong et al., 2015), and the mesolimbic pathway (Oleson & Cheer, 2012). Given its key role in dopamine metabolism, especially in the prefrontal cortex, COMT has been suggested to be a good candidate for gene‐environment interaction effects in psychosis, such as with cannabis (Bilder, Volavka, Lachman, & Grace, 2004).

The association of rs4680 with psychosis phenotypes in the context of cannabis use has been difficult to replicate (Okochi et al., 2009; Zammit, Owen, Evans, Heron, & Lewis, 2011; Zammit et al., 2007). This may be due to heterogeneity between studies, including differences in age of assessment of psychotic symptoms and variability in the allelic frequency of rs4680 between different ethnic groups including apparently similar ethnic groups with differing degrees of admixture. An important factor could be that the relationship between rs4680, cannabis, and psychosis is complicated by rs4680 being associated not only with psychosis phenotypes but also with cannabis use. A study reported higher frequency of the rs4680 Val/Val genotype in individuals with schizophrenia who had used cannabis premorbidly (88.9%) compared to those who had not (68.4%) (Ermis et al., 2015). However, the allelic effect is not consistent in the literature: An earlier study had observed higher cannabis use in *COMT* Met/Met homozygotes compared to Val/Val homozygotes (Costas et al., 2011). Indeed, in a study of genetic etiology more broadly, there is evidence of common genetic predisposition to schizophrenia and to the risk of cannabis use (Power et al., 2014). Owing to this, schizophrenia and cannabis use may be both associated and correlated with each other. In our study, we sought to reexamine the effect of *COMT* rs4680 genotype on onset of psychosis, adjusting for relevant covariates such as cannabis use, in a Canadian Caucasian sample. Our hypothesis was that rs4680 Val/Val genotype would confer an earlier AoP, adjusting for relevant covariates including cannabis use.

## 2 METHODS

### 2.1 Sample

Recruitment was done from a variety of locations. These included two first‐episode psychosis teams, one in Edmonton, Alberta (the Edmonton Early Psychosis Intervention Clinic, EEPIC), and one in Halifax, Nova Scotia (the Nova Scotia Early Psychosis Program, NSEPP). In addition, patients were recruited from Alberta Hospital Edmonton (Neuropsychology Department), and from various Halifax community mental health teams. A total of 52.66% were minimally medicated or in the early stages of their illness and 47.34% were medicated for more than 3 months. The mean duration of illness for the minimally medicated and medicated for more than 3 month groups were 0.78 (95% CI 0.507–1.054) and 9.37 (95% CI 7.213–11.545) years, respectively. Research ethics committee approval was obtained at both sites as part of a study investigating genetic associations with psychotic disorders, and all patients provided informed consent. Patients with the following DSM‐IV diagnoses (made using the Structured Clinical Interview for DSM‐IV, or SCID‐I) were included: schizophrenia, schizoaffective disorder, schizophreniform disorder, delusional disorder, brief psychotic episode, psychosis not otherwise specified, and substance‐induced psychosis. AoP in our study was defined as age in years when DSM diagnostic criteria were met. This was determined retrospectively from the time of intake through interviews and medical records. A self‐rated drug screen in the form of a computerized questionnaire was used to gather information on substance use including lifetime cannabis use and age at first usage (Purdon, 2007). The latter was collected using the following categories: less than 11 years, 11–15 years, 16–19 years, 20–29 years, 30–39 years, and above 40 years. These were collapsed into never used cannabis, first use of cannabis before 20 years (the first three categories, equal to 19 years and under), and first use of cannabis at or after 20 years (the last three categories, which will be referred to as first cannabis use after 20 years).

DNA was extracted from saliva collected using Oragene DNA collection kits (DNA Genotek Inc., Ottawa, ON, Canada). The earlier part of the collection was extracted in the University of Alberta Applied Genomics Core (TAGC) on a Beckman Biomek NX‐automated workstation, with the latter part of the sample being extracted manually according to the manufacturer's instructions with a minor modification. For the former, 0.6 ml of saliva/Oragene buffer was processed using the Agencourt GenFind v2 kit, without initial lysis (as a 50°C incubation of sample with the lysis buffer preceded loading on to the instrument), and in the initial step, two washes with 75% ethanol were performed prior to elution of the genomic DNA in 55 μl of elution buffer. For the latter, the Invitrogen PureLink Genomic DNA mini kit protocol was used with minor modifications. In brief, 2 ml of the saliva/buffer mixture was processed. Genomic DNA was precipitated, resuspended in phosphate‐buffered saline and then bound to a filter on a spin column. The column was washed twice with 60%–70% ethanol, and the DNA was eluted twice; the first time with 100 μl of prewarmed elution buffer, the second time with 50 μl prewarmed elution buffer. Both elutions were then transferred to sterile cryovials for storage at −80°C, in which the samples were encoded using laboratory numbers with no personal identifiers.

### 2.2 Genotyping

Genotyping was initially conducted using a SNaPshot assay in The Applied Genomics Centre (TAGC) at the University of Alberta, with this being continued in the Aitchison laboratory by a TaqMan^®^ SNP Genotyping Assay (ID: C__25746809_50) on an Applied Biosystems ViiA™ 7 Real‐Time PCR System (ThermoFisher Scientific, Canada, formerly Applied Biosystems by Life Technologies, Canada). DNA fragments for use in the SNaPshot reaction were generated by polymerase chain reaction (PCR) using the following conditions: 95°C for 5 min; 32 cycles of 95°C for 30 s, 65°C for 90 s, and 72°C for 30 s; with a final extension at 68°C for 10 min. PCR template (0.056 pmols) was added to 2 μl of SNaPshot multiplex ready reaction mix and 3 μl of 2.5× BigDye (ABI, USA) sequencing buffer. The SNaPshot conditions for primer extension were as follows: 25 cycles of 96°C for 10 s, 50°C for 5 s, and 60°C for 30 s. Primer‐extended products were treated with shrimp alkaline phosphatase (SAP), denatured at 98°C for 3 min, and chilled on ice for 3 min before processing on an AB3130 Genetic Analyzer (ThermoFisher Scientific, Canada, formerly Applied Biosystems by Life Technologies, Canada). Data were analyzed using Applied Biosystems GeneMapper v 4.0. For the TaqMan assay, all samples were genotyped in duplicate, with an in‐house automated data comparison to compare genotypes between duplicates. Repeats were conducted for any calls not readily resolved.

### 2.3 Population stratification analysis

Given the differential allelic frequency of rs4680 by ethnic group, we restricted the analysis to Caucasians (strictly defined using all available data from the grandparent level). We ensured that there were no ethnic variations by genotyping 24 markers with known allele frequencies in Caucasians (Kosoy et al., 2009). Data for one of these did not pass quality control; for the remaining 23, the minor allele frequencies did not significantly differ from those expected in Caucasians (*p *=* *.35).

### 2.4 Statistical analysis

Statistical analysis was conducted using STATA 13.1. The distribution of demographic variables and other results were compared by chi‐square or *t*‐tests for categorical and linear variables, respectively. Kaplan–Meier time‐to‐event analyses were performed using the log‐rank “sts test” with AoP as a continuous variable, entering gender, age at first cannabis use categories (before vs. on or after 20 years), and rs4680 genotype (Val/Val, Val/Met, and Met/Met) as predictors. Kaplan–Meier analysis was repeated in subgroups of interest.

## 3 RESULTS

One hundred and sixty‐nine individuals met diagnostic criteria and had data available on AoP, cannabis use, and *COMT* rs4680 genotype. There was 100% concordance between the data from TAGC and the Aitchison laboratory, and all samples except one were genotyped by both methods. The genotypes were in Hardy–Weinberg equilibrium (χ^2^ = 0.4095, *p *=* *.52), with 0.47 and 0.53 being the allele frequencies for the Val and Met, respectively. Lifetime cannabis use was more common among males (*p* = .004), and first cannabis use under 20 years of age was more common in males (*p* = .01) (Table 1).

**Table 1:** Sample characteristics by cannabis use categories (*p* values by chi‐squared analysis or *t*‐test)

	Sample	First cannabis use			*p*
		Never users	Before 20 years	After 20 years	
*N*	169	24	127	24	
Gender (*N*, %)					
Male	119	11 (45.83)	95 (76.00)	15 (65.0)	**.01**
Female	50	13 (54.17)	30 (24.00)	7 (35.0)	
Diagnosis, *N* (%)					
Schizophrenia spectrum disorder	109	18 (75)	80 (64.00)	11 (55.0)	.282
Psychosis NOS	37	6 (25)	26 (20.80)	5 (25.00)	
Substance‐induced psychosis	23	0 (0)	19 (15.20)	4 (20.00)	
Education (*N*, %)					
Grade 12 or less	61	6 (25)	53 (42.00)	2 (10.0)	**.009**
Relationship status (*N*, %)					
Single	156	22 (91.67)	117 (93.60)	17 (85.0)	.404
Married/been with partner	13	2 (8.33)	8 (6.40)	3 (15.0)	
Mean AoP (years, *SD*)	22.86 (6.38)	23.33	22.12	26.92	**.006**
Mean age (years)	27.71	32.58	26.25	31.01	**.003**

AoP, Age of onset of psychosis. P values <0.05 in bold.

**Figure 1:**
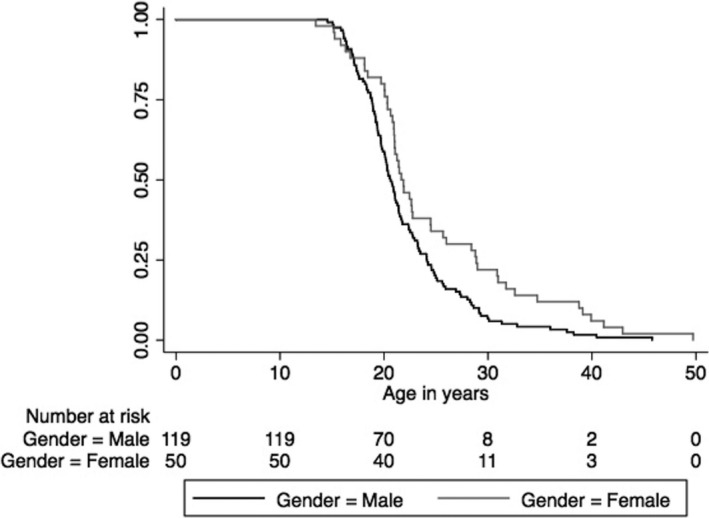
Time to age of diagnosis of psychosis by gender and *COMT* Val158Met genotype

On Kaplan–Meier time‐to‐event analysis, male subjects had an earlier AoP than females (median AoP: males = 20.61 years, females = 21.66 years, log‐rank test *p *=* *.0086, Figure 1). The main effect of rs4680 in the time‐to‐event analysis was not significant (although the pattern of the median AoPs was Val/Val < Val/Met < Met/Met, 19.70, 20.96 and 21.90 years, respectively; log‐rank test *p *=* *.251). In those who had used cannabis, first use of cannabis prior to 20 years of age was associated with earlier AoP (*p *=* *.005).

Given previous findings, we explored the effect of rs4680 genotype in those who had first used cannabis at less than 20 years of age; in these, the association between rs4680 genotype and AoP was close to significant, with patients of Val/Val genotype developing psychosis earliest (median AoP: Val/Val < Val/Met < Met/Met 19.37, 20.95, 21.24 years, respectively; log‐rank test *p *=* *.051). On repeating this stratifying by gender, a trend‐level association remained (log‐rank test *p *=* *.079).

## 4 DISCUSSION

In summary, although our data did not indicate a significant effect, there was a trend‐level signal in the same direction as some previous studies (Estrada et al., 2011) that examined rs4680, specifically that Val/Val genotype may be associated with an earlier AoP, in those who had first used cannabis relatively early in life (prior to 20 years). STATA provides the option of statistically testing for the trend of survivor functions and when we used the test for the trend of rs4680 on AoP in first cannabis users before 20 years of age, it was significant (*p *=* *.029). The pattern of the trend is consistent with an additive pattern of genotype effect (Val/Val > Val/Met > Met/Met), consistent with the codominant pattern described for these alleles.

These results echo the investigation of rs4680 in the Dunedin birth cohort study, with the Val/Val genotype being the at risk genotype for a psychotic disorder in Caucasians (Caspi et al., 2005). Our study had similarities to the Dunedin study, for example, the majority of our patients had a schizophrenia spectrum disorder, and the association described in the former was with schizophreniform disorder. Another likely similarity is the ethnicity of Canadians and the New Zealanders, and therefore the genetic background upon which the *COMT* rs4680 variant sits and with which it may interact to determine overall expression of the COMT enzyme (Tunbridge, 2010). As described above, we have also confirmed ethnic homogeneity.

The usefulness and contributions of studies that the examine the effect of genetic variants in the context of environmental factors in psychiatry has been questioned (Zammit, Owen, & Lewis, 2010) and part of the reason for this is nonreplication of earlier results. In the case of the moderation by rs4680 of the association between cannabis and psychosis, although a number of studies have been done, these vary in: the population in which studies were conducted (e.g., normal subjects or those with psychosis), the definition of psychotic disorder or of psychotic phenomena, the use of DSM for diagnostic criteria, the length of follow‐up of patients, the definition of cannabis use or misuse, and the ethnicity of participants. We included substance‐induced psychosis in our definition of psychosis and while this may theoretically introduce some genetic heterogeneity, there are studies that indicate that primary and drug‐induced psychosis may be genetically linked (Bramness et al., 2012). In our opinion, our study has many similarities with that of Dunedin study and hence the consistency in findings. *COMT* remains an interesting candidate gene for gene‐environment interactions in psychosis since it modulates dopamine function, is dynamically regulated and its expression alters with environmental stimuli (Tunbridge, 2010). In psychosis, the Val/Val rs4680 genotype may predispose individuals to stress‐related mesolimbic hyperactivity (Ira et al., 2014) and is known to increase the vulnerability to psychosis after cannabis use in those exposed to childhood abuse (Alemany et al., 2014). rs4680 has also been reported to moderate the effect of stress in induction of psychotic symptoms in a study of army recruits (Stefanis et al., 2007).

In our sample, the effect of age at first use of cannabis before 20 years on the AoP was significant. This is consistent with earlier examinations of the cannabis–psychosis relationship (Andreasson, Allebeck, Engstrom, & Rydberg, 1987; Arseneault, Cannon, Witton, & Murray, 2004; Arseneault et al., 2002; Compton et al., 2009; Di Forti et al., 2009, 2014; Kelley et al., 2016; McLaren, Silins, Hutchinson, Mattick, & Hall, 2010; Moore et al., 2007) (for a meta‐analysis, see Large, Sharma, Compton, Slade, & Nielssen, 2011). The effect of gender on AoP was significant in our study, with males being at risk of developing psychosis earlier than females. This is consistent with prior studies (Castle, Sham, & Murray, 1998; Hafner et al., 1994), especially for schizophrenia spectrum disorders. In a review of pertinent data, it has been suggested that where such an effect has not been found, factors such as atypical marital status and premorbid personality may contribute (Jablensky & Cole, 1997). Of note, 92% of our patients were single and only 2.86% were married, which is as expected for schizophrenia spectrum disorders (the majority of our sample). It is therefore not surprising that our sample demonstrated the same effect of gender on age of onset as the majority of schizophrenia spectrum samples that have been studied to date.

### 4.1 Strengths and limitations

Strengths of this study include defining psychotic disorder by structured clinical interview for DSM‐IV diagnosis and careful definition of Caucasian ethnicity. The main limitations of the study were the relatively small sample size for a genetic association analysis, especially that of the controls, the self‐report nature of the cannabis data and collection of first cannabis use age data in the form of age ranges. It is worth noting, however, that the time‐to‐event analysis for rs4680 was sufficiently powered to detect significant effects. We calculated the power of our sample using the “stpower logrank” command in STATA, which uses the Freedman method (Freedman, 1982) to estimate sample sizes for a two sample comparison of survivor functions. Based on a one‐sided 0.05 significance level log‐rank test, a ratio of 1:3 for the frequency of the Val/Val genotype versus the rest (as seen in our data), a 50% reduction in hazard ratio (as seen in our sample for Val/Val group compared to the rest), to achieve power of 0.8, a sample size of 104 patients with only 73 with onset of psychosis was required. Hence, with our sample of 175 subjects, we were sufficiently powered to detect the effect of the genotype. Forty‐nine volunteers without psychosis, of a similar age but with different gender and cannabis use patterns to the psychosis subjects and with cannabis data were recruited and genotyped in parallel as controls for this genetic study. Of note, when these controls were added to the time‐to‐event analysis, we observed that the association between rs4680 and AoP in early cannabis users now reached significance even after adjustment for gender (median AoP: Val/Val < Val/Met < Met/Met 19.37, 21.48, 22.34 years, respectively; log‐rank test *p *=* *.0243). The self‐report nature of the cannabis use data could also be viewed as a limitation; it would be useful to supplement this with additional material from interviews and medical record documentation in the future. It is unlikely, however, that rs4680 genotype would have exerted a biasing effect on an individual's self‐reported cannabis use history.

### 4.2 Future directions

Independent replication in another Caucasian sample is clearly desirable and intended. In addition, extension of this to include childhood trauma data is desirable (Roper, Purdon, & Aitchison, 2015). This could be combined with epigenetic analysis of, for example, the *COMT* promoter, methylation of which has been shown to be associated with frequency of cannabis use in adolescents and young adults (van der Knaap et al., 2014). We suggest that future studies should examine the relationship between age of onset of prodromal symptoms, duration of untreated psychosis, age at first presentation and age of onset of DSM symptoms, with *COMT* genotyping and thorough cannabis data collection, preferably in the same sample and in controls in parallel, to enhance the understanding of these effects.

In summary, our study shows that, in those who used cannabis before age 20 years, having the rs4680 Val/Val genotype could be associated with the earliest age of diagnosis of psychosis, with the Val/Met having a slightly later age of diagnosis and the Met/Met still later. The earlier the illness commences, the more crucial social transitions (such as completing educational and vocational training, independent living, and partner selection) may be interrupted. With some genetically vulnerable individuals, cannabis use may be associated with a lasting schizophrenia type of psychotic illness.

## DECLARATION OF INTEREST

KJA reports consultancy services for Otsuka Canada Pharmaceutical Inc., and Lundbeck Canada SEP has received honoraria for speaking, advisory board consultation, and contracted services from Merck Pharma and Lundbeck Canada, and an investigator‐initiated operating grant from the Zyprexa Research Foundation of Eli Lilly Canada, plus royalties from sales of the Screen for Cognitive Impairment in Psychiatry – Spanish language version (SCIP‐S). PT has been in Advisory Boards and/or received speaker fees over the last 12 months from Janssen Inc., Otsuka Canada Pharmaceutical Inc. and Roche (Canada).
