# Investigation of the *COMT* Val158Met variant association with age of onset of psychosis, adjusting for cannabis use

**DOI:** 10.1002/brb3.1120

**Published:** 2018-09-27

**Authors:** Rohit J. Lodhi, Yabing Wang, David Rossolatos, Georgina MacIntyre, Alexandra Bowker, Candice Crocker, Hongyan Ren, Aleksandra Dimitrijevic, Darren A. Bugbee, Alexandra Loverock, Brett Majeau, Sudhakar Sivapalan, Virginia M. Newton, Philip Tibbo, Scot E. Purdon, Katherine J. Aitchison

In Lodhi et al. (2017), the following errors were published:

- In the Abstract, the words “Logistic regression and” in the last sentence of “Methods” section should have been removed so it reads “Kaplan–Meier analysis results are reported.”
- In Figure 1, the two rows of numbers in graph stating “Number at risk, Gender = male, Gender = female” were incorrectly labeled and not transparent to the reader. And the authors would like to correct and simplify the caption from “Time to age of diagnosis of psychosis by gender and *COMT* Val158Met genotype” to “Time to age of diagnosis of psychosis by gender.”

The correct Figure 1 is shown below:

**Figure 1:**
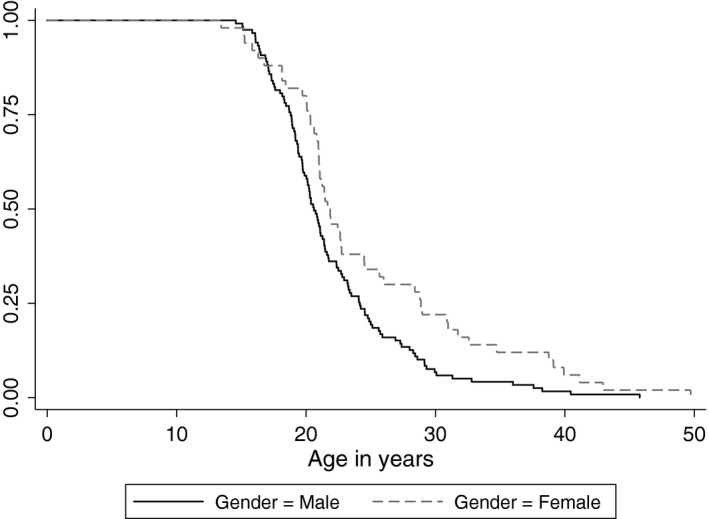
Time to age of diagnosis of psychosis by gender
